# Harnessing piezoelectricity for bone tissue engineering: recapitulating the electrophysiological microenvironment through smart biomaterials

**DOI:** 10.3389/fbioe.2026.1867122

**Published:** 2026-06-29

**Authors:** Bo Shao, Yu He, Guiyun Wang, Zhuang Liu, Suqin Wang

**Affiliations:** 1 College of Engineering, Shandong Xiehe University, Jinan, China; 2 College of Nursing, Shandong Xiehe University, Jinan, China; 3 School of Materials Science and Engineering, Shandong University, Jinan, China

**Keywords:** bone tissue engineering, electrophysiological microenvironment, osteogenesis, piezoelectric biomaterials, wireless actuation

## Abstract

Natural bone tissue is a highly dynamic electrophysiological microenvironment, where endogenous piezoelectricity plays a pivotal role in regulating bone remodeling and homeostasis. Addressing the complex clinical challenges of bone defects, piezoelectric biomaterials have emerged as a revolutionary self-powered therapeutic strategy, reconstructing this critical biophysical niche through electromechanical conversion. This article systematically reviews the classification and characteristics of inorganic ceramics, organic polymers, composites, and emerging systems. Advanced energy conversion strategies, ranging from physiological motion to low-intensity pulsed ultrasound actuation, are critically evaluated. Furthermore, the multi-scale mechanisms involving ion channel activation, cellular energy metabolism reprogramming, osteoimmune microenvironment remodeling, and osteogenic gene transcription and mineralization are elaborated. Ultimately, this review aims to provide profound theoretical guidance for the clinical translation of next-generation smart orthopedic implants.

## 1 Introduction

Bone is a highly mineralized and metabolically active connective tissue that possesses exceptional weight bearing, supporting, and microcirculatory functions (Buck and Dumanian, 2012; Florencio-Silva et al., 2015). Its specific morphological and functional characteristics enable it to continuously provide structural support, protection, and remodeling through a delicate balance between osteoclast mediated resorption and osteoblast mediated formation (Kim J.-M. et al., 2020; Chen et al., 2018a). Although bone tissue possesses an intrinsic regenerative capacity, bone defects caused by trauma, tumor resection, or infection often exceed its self-healing potential and remain a major clinical challenge (Migliorini et al., 2021; Kanczler and Oreffo, 2008). Currently, the gold standard for treating such defects remains autologous or allogeneic bone grafting (Fernandez de Grado et al., 2018; Calori et al., 2011). However, this approach faces significant limitations, including restricted tissue availability and secondary trauma at the donor site.

Since the 19th century, the scientific community has recognized that bone structure responds to mechanical forces, a concept famously known as Wolff’s Law (Bassett, 1967). However, the physical mechanism behind this law was not revealed until the discovery of the piezoelectric effect in bone in 1950s (Fukada and Yasuda, 1957). Bone piezoelectricity refers to the ability of bone to generate electrical signals under mechanical stress, which originates from the noncentrosymmetric arrangement of collagen fibers and hydroxyapatite within its structure (Isaacson and Bloebaum, 2010; Kamel, 2022). Under the mechanical stress generated by daily physiological activities such as walking, the sliding and deformation of collagen fibers produce a macroscopic piezoelectric potential (Fukada and Yasuda, 1964; Ahn and Grodzinsky, 2009). For example, the human tibia can generate a piezoelectric potential of approximately 300 μV during normal walking (YASUDA, 1977). These stress induced microcurrents and negative charges can guide the migration of mesenchymal stem cells, enhance osteoblast activity, and promote matrix mineralization, thereby playing a crucial role in driving bone formation and remodeling (JAHN, 1968; Khare et al., 2020; Rajabi et al., 2015).

To accelerate the repair of bone injuries, exogenous electrical stimulation therapies have long been employed in clinical settings (Yao et al., 2025; Zhang et al., 2023; Sun et al., 2025). Since the 1980s, electrical stimulation has become an effective adjunctive therapy by mimicking the endogenous electric field to stimulate osteoblast activity (NODA and SATO, 1985; Ferrier et al., 1986). However, traditional electrical stimulation devices are typically bulky, require frequent battery replacements, and are difficult to integrate seamlessly with biological tissues, which severely restricts their long-term clinical application. In this context, piezoelectric biomaterials have emerged as a revolutionary self-powered therapeutic strategy (Zhang Z. et al., 2025; Ni et al., 2025). Piezoelectric materials can convert endogenous or exogenous mechanical energy into localized electrical signals to reconstruct the electrophysiological microenvironment of natural bone (Damjanovic, 1998). Ranging from inorganic piezoelectric ceramics such as barium titanate and zinc oxide to organic piezoelectric polymers like polyvinylidene fluoride (PVDF) and poly-L-lactic acid (PLLA), as well as emerging piezoelectric hydrogels and nanocomposites, these smart materials offer abundant choices for constructing advanced bone tissue engineering scaffolds (Jacob et al., 2018; Chen et al., 2025).

Compared to traditional passive scaffolds, piezoelectric scaffolds can provide active biophysical induction. They trigger intracellular biochemical cascades by activating voltage gated calcium channels and mechanosensitive channels like PIEZO1 (Wang et al., 2020; Jiang T. et al., 2024; Liu et al., 2022a), significantly upregulating the expression of core osteogenic genes such as runt-related transcription factor 2 (RUNX2) (Zhang et al., 2018; Liu Q. et al., 2022; Bai et al., 2023) and bone morphogenetic protein-2 (BMP-2) (Song et al., 2025). Furthermore, the piezoelectric effect can regulate the local immune microenvironment by promoting the polarization of macrophages toward a regenerative M2 phenotype and scavenging excess reactive oxygen species, thereby establishing a favorable niche for tissue regeneration (Wu et al., 2023; Wang L. et al., 2025; Zheng H. et al., 2025). To achieve precise bone repair, researchers have developed various energy conversion strategies. While early studies focused on utilizing endogenous power like walking or muscle contraction, employing external energy fields such as low intensity pulsed ultrasound (LIPUS) for remote and noninvasive actuation has become the current research frontier, especially for severely traumatized patients with limited mobility. Ultrasound actuation not only provides exceptional deep tissue penetration but also generates continuous electrical stimulation through acoustoelectric conversion to achieve true *in situ* energy harvesting.

Although research on piezoelectric bone repair materials is continuously increasing, a systematic bridging between the underlying biophysical actuation mechanisms and downstream osteogenic signaling cascades is still required. This review aims to systematically explore the applications of piezoelectric biomaterials in bone tissue engineering. First, the piezoelectric origins and electrophysiological characteristics of natural bone are described in detail. Next, based on chemical composition, structural dimensions, and state of matter, inorganic ceramics, organic polymers, and composite materials are discussed and categorized. Subsequently, we highlight the architectural transition toward frontier structural formulations and emerging piezoelectric systems, including tissue-compliant hydrogels, low-dimensional nanomaterials, and smart programmable scaffolds. This article then critically evaluates advanced wireless actuation strategies for these piezoelectric biomaterial systems. This article then critically evaluates advanced actuation strategies for piezoelectric scaffolds. Next, the profound mechanisms by which piezoelectric signals promote osteogenesis are elaborated. This includes the initial alteration of cell membrane potentials and the activation of ion channels, followed by the reprogramming of cellular energy metabolism, the remodeling of the osteoimmune microenvironment, and the transcription and mineralization of osteogenic genes. By comprehensively mapping the entire pathway from dipole electromechanical conversion to macroscopic tissue regeneration, this review aims to provide profound theoretical guidance for the clinical translation of advanced smart orthopedic implants.

## 2 Endogenous piezoelectric effect and electrophysiological microenvironment in natural bone tissue

Natural bone tissue is a highly complex and metabolically active composite structure, primarily comprising an extracellular matrix (ECM) and various cellular components (Wang et al., 2022). The ECM consists of an inorganic mineral phase and an organic matrix (Weiner and Wagner, 1998). The inorganic phase, accounting for approximately 65% of the total bone mass, is predominantly composed of nano-hydroxyapatite (HAp) crystals that endow the bone with essential mechanical strength and rigidity. The organic matrix, which constitutes about 25%–30% of its mass, is overwhelmingly dominated by type I collagen fibers, providing crucial flexibility and tensile elasticity. Interspersed within this matrix are specialized cellular components, including osteoblasts responsible for matrix secretion, osteoclasts that mediate bone resorption, and osteocytes that orchestrate bone microenvironment homeostasis. It is precisely this exquisite composite architecture, characterized by the integration of organic collagen fibers and inorganic HAp crystals, that not only provides mechanical support but also intrinsically endows natural bone with unique piezoelectric properties.

Beyond its static mechanical role, natural bone serves as a highly dynamic electrophysiologically active microenvironment. Fundamentally, the electrophysiological microenvironment in bone tissues is defined as a highly integrated, dynamic biophysical niche comprising synchronized electrical cues from both solid and liquid phases. Rather than representing an isolated electrical property, it encompasses the cooperative interaction of solid-phase piezoelectric charge generation (from collagen and hydroxyapatite deformation), liquid-phase streaming potentials (driven by interstitial fluid flow), and the resulting localized potential differences. These coupled fields generate precise ion fluxes that continuously perturb the cellular boundary, directly modulating transmembrane ion channel activity, altering cellular resting membrane potentials, and regulating bioenergetic states to sustain skeletal homeostasis and drive tissue repair.

**FIGURE 1:**
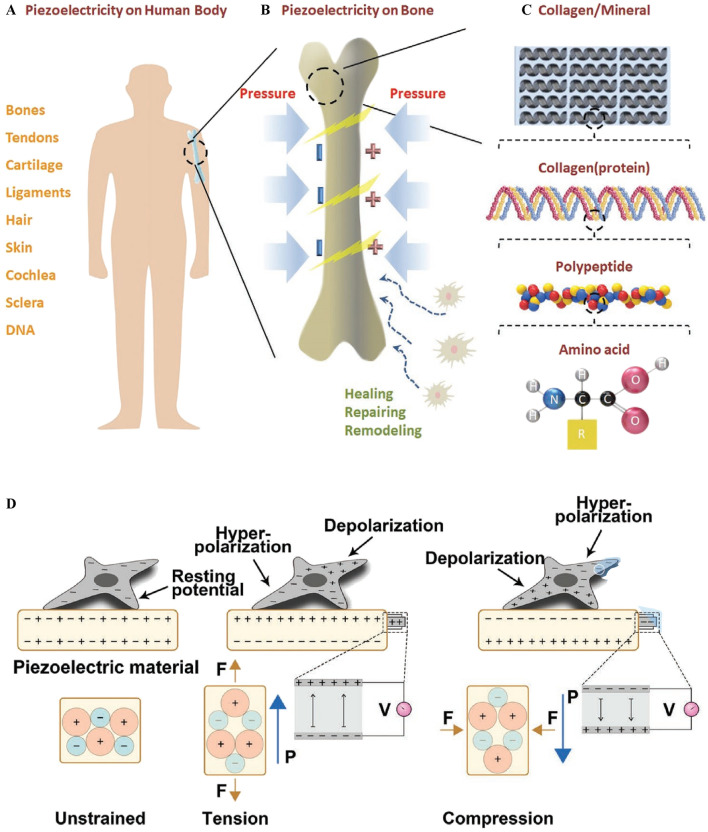
**(A)** Mapping the distribution of piezoelectric properties across various physiological tissues within the human body. Reproduced with permission (Kim D. et al., 2020). Copyright 2020, Wiley-VCH. **(B)** Role of endogenous piezoelectric potentials in bone-specific cellular regeneration. Reproduced with permission (Kim D. et al., 2020). Copyright 2020, Wiley-VCH. **(C)** Structural levels of piezoelectric bone, comprising collagen fibrils and their constituent peptides and amino acids. Reproduced with permission (Kim D. et al., 2020). Copyright 2020, Wiley-VCH. **(D)** Mechanism of strain-induced electrical charge generation on piezoelectric matrices and its impact on cell signaling. Reproduced with permission (Kapat et al., 2020). Copyright 2020, Wiley-VCH.

First, the inherent piezoelectricity of bone was first discovered by Yasuda and Fukada in the 1950s (Fukada and Yasuda, 1957), providing a profound biophysical explanation for Wolff’s law, which states that bone adapts its internal architecture in response to external mechanical loads. Piezoelectricity has been identified in a diverse array of biological materials across nature and the human body, including bone, tendon, skin, hair, wood, clamshell, viruses, and fundamental molecules such as DNA, nucleotides, and amino acids (Figure 1A). The piezoelectric coefficient of human tibia was measured to be up to 8.7 pC/N at the nanoscale with a piezo-response force microscope (PFM) (Halperin et al., 2004) (Figure 1B). The primary source of bone piezoelectricity is the highly oriented type I collagen fibrils within the organic matrix (Minary-Jolandan and Yu, 2009) (Figure 1C). Collagen molecules possess a non-centrosymmetric triple-helix polypeptide structure stabilized by hydrogen bonds. When mechanical stress is applied, the sliding and deformation of these collagen fibers lead to the distortion of cross-linking hydrogen bonds and the displacement of internal charge centers, thereby generating a macroscopic piezoelectric potential (Ahn and Grodzinsky, 2009; Halperin et al., 2004). In addition to collagen, HAp also significantly contributes to the overall bioelectricity of bone due to its non-centrosymmetric monoclinic crystal phase (Pérez-Solis et al., 2018; Han et al., 2021). Furthermore, the interface between collagen fibrils and HAp crystals can function analogously to a semiconducting P-N junction, where shear stress caused by mismatched elastic moduli facilitates localized charge generation and separation (BECKER and BACHMAN, 1965; Hastings and Mahmud, 1988). Besides, under authentic physiological conditions, the electromechanical response is further modulated by fluid dynamics. Mechanical loading drives the flow of interstitial fluid, which is rich in ions, through the canalicular-lacunar network, thereby generating a streaming potential (Walsh and Guzelsu, 1993). This liquid-phase streaming potential dynamically couples with the solid-phase piezoelectric effect to establish a comprehensive electrophysiological microenvironment (Rajabi et al., 2015; Liu et al., 2021a). Finally, membrane potential regulation occurs as cellular resting membrane potentials are altered which subsequently activates voltage gated and mechanosensitive ion channels. Collectively these integrated components establish a sophisticated biophysical niche that continuously coordinates bone homeostasis structural adaptation and tissue regeneration.

Fundamentally, these endogenous electrical signals serve as the biophysical foundation of Wolff’s Law, dictating that bone structurally adapts to mechanical loads. Despite these micro-currents are weak (typically within 10 µA/cm^2^), both the electrical potential difference and micro-current play a crucial role in the bone repair process. Upon mechanical deformation, the resulting piezoelectric charges establish a differential electrophysiological microenvironment: the compressed (concave) side becomes electronegative, recruiting osteoblasts to promote bone formation, whereas the tensed (convex) side becomes electropositive, attracting osteoclasts to induce bone resorption (Bassett et al., 1964). At the cellular level, this electromechanical transduction alters the resting cell membrane potential, triggering the activation of voltage-gated calcium channels (VGCCs) (Pall, 2013), mechanosensitive channels (e.g., PIEZO1) (Zhou et al., 2020), and transient receptor potential vanilloid four channel (TRPV4) (O’conor et al., 2014). The subsequent massive influx of extracellular Ca^2+^ activates the calmodulin/calcineurin pathway, promoting the nuclear translocation of NF-AT (Figure 1D) (Jacob et al., 2018; Kapat et al., 2020; More and Kapusetti, 2017). Synergizing with MAPK/ERK and Wnt/*β*-catenin cascades, these signals dramatically upregulate the expression of core osteogenic transcription factors and proteins, such as RUNX2 and BMP-2, instructing osteoblasts to accelerate extracellular matrix synthesis. Concurrently, the endogenous electric field drives the electrophoretic enrichment of interstitial Ca^2+^ and PO_4_^3-^ ions (Kubo, 2012; Lacroix and Prendergast, 2002), thereby facilitating the targeted nucleation and directed crystallization of hydroxyapatite.

## 3 Piezoelectric biomaterials: classification and characteristics

**FIGURE 2:**
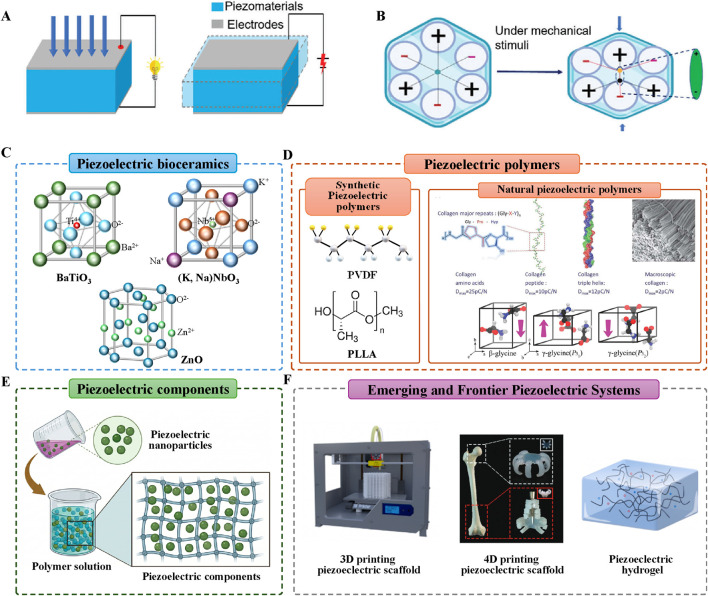
**(A)** Piezoelectric effect and reverse piezoelectric effect. Reproduced with permission (Zhang J. et al., 2025). Copyright 2025, Wiley-VCH. **(B)** Mechanism of piezoelectric potential generation in a crystal (Ghosh et al., 2022). **(C)** Common types of piezoelectric biological ceramics. **(D)** Common piezoelectric polymers include synthetic piezoelectric polymers and natural piezoelectric polymers. Reproduced with permission (You et al., 2021). Copyright 2019, MDPI. Reproduced with permission (Liu et al., 2020). Copyright 2018, Royal Society of Chemistry. **(E)** The synthesis method of piezoelectric composites. **(F)** Emerging and frontier piezoelectric systems. Reproduced with permission (You et al., 2021). Copyright 2021, Wiley-VCH.

Piezoelectric biomaterials are recognized for their exceptional capacity to convert mechanical stress into electrical energy, thereby providing a highly biomimetic electromechanical microenvironment that is critical for bone tissue engineering. Piezoelectricity is defined as the electric polarization in a substance in response to the application of mechanical stress (Zhang J. et al., 2025) (Figures 2A,B). The linear conversion of mechanical energy to electrical energies, which is given by Equation 1, is a property typical of non-symmetric substances (Damjanovic, 1998).

$$D=dE+\varepsilon^{T}E$$ (1)

where *D* is the dielectric displacement (consider it equal to polarization), *T* the stress, *E* the electric field, and *ε* the dielectric constant (permittivity) (Haertling, 1999). To accurately mimic the endogenous piezoelectricity of natural bone and meet the multifaceted requirements of implantation, a diverse array of materials has been developed. Based on their chemical composition, structural dimensions, and state of matter, these materials can be broadly classified into four major categories: inorganic piezoelectric ceramics, organic piezoelectric polymers, and piezoelectric composites, as shown in Figures 2C–E.

### 3.1 Inorganic piezoelectric ceramics

Inorganic piezoelectric ceramics typically exhibit superior piezoelectric coefficients and excellent electromechanical coupling efficiency, rendering them capable of delivering robust electrical stimulation. The paramount concern for their application in bone tissue engineering is biosafety. To avoid the heavy metal toxicity associated with lead-based ceramics (e.g., PZT), research has shifted toward several biocompatible lead-free systems, such as barium titanate (BaTiO_3_, BT), potassium sodium niobate (K_0.5_Na_0.5_NbO_3_, KNN), and zinc oxide (ZnO), and so on (Figure 2C).

BT is a prototypical lead-free perovskite ceramic and one of the most extensively investigated materials in orthopedics due to its extremely high piezoelectric coefficient (up to ∼190 pC/N) and favorable cytocompatibility. Below its Curie temperature of approximately 120 °C, BT undergoes a phase transition from a symmetric cubic phase to an asymmetric tetragonal structure, wherein the relative displacement of Ti^4+^ generates spontaneous polarization and extremely high surface piezoelectric potentials. Despite its excellent cytocompatibility, the high rigidity and non-degradable nature of barium titanate often restrict its use as a bulk implant. Consequently, it is frequently employed as an active coating on porous metallic implants, such as 3D-printed Ti-based scaffolds, where mechanical loads or LIPUS can activate the piezoelectric effect to promote anti-inflammatory M2 macrophage polarization, angiogenesis, and rapid osseointegration (Wu et al., 2023; Liu et al., 2020; Fan et al., 2020). Recent frontier applications further endow BT with multi-functionality *via* elemental doping. For instance, calcium and manganese co-doping allows for the sustained release of osteogenic ions while enhancing piezoelectric output (Zheng et al., 2021). Moreover, sulfur doping introduces oxygen vacancies that significantly enhance its piezocatalytic generation of reactive oxygen species (ROS) for antibiotic-free eradication of bacterial biofilms under ultrasound irradiation (Lei et al., 2022).

ZnO represents another highly promising lead-free inorganic piezoceramic, distinguished by its non-centrosymmetric wurtzite crystal structure. In this tetrahedral coordination, the spatial separation of zinc and oxygen atoms inherently endows ZnO with native piezoelectricity without the necessity for high-voltage poling, yielding a piezoelectric coefficient (*d*_33_) of approximately 12.4 pC/N (Crisler et al., 1968). Beyond its electromechanical coupling, ZnO exhibits exceptional multifunctionality highly beneficial for bone tissue engineering (Li et al., 2020). The controlled degradation of ZnO releases Zn^2+^ ions, which serve as crucial trace elements that simulate insulin-like effects to significantly promote osteogenic differentiation, extracellular matrix mineralization, and angiogenesis. Furthermore, under mechanical or ultrasound stimulation, ZnO demonstrates potent piezocatalytic properties, generating ROS that effectively disrupt bacterial membranes, thereby conferring robust, antibiotic-free antibacterial capabilities. To mitigate potential dose-dependent cytotoxicity and harness its full potential, ZnO is frequently engineered into nanostructures and incorporated into biodegradable polymeric matrices like polycaprolactone (PCL) or chitosan (Khader and Arinzeh, 2020). This composite strategy not only ensures the sustained and safe release of zinc ions but also synergistically enhances the mechanical integrity and piezoelectric output of the scaffolds, creating an optimal electrophysiological microenvironment for accelerating bone regeneration.

KNN is a prominent lead-free piezoceramic characterized by a high Curie temperature (*T*_C_ = 420 °C), low dielectric constant, and robust electromechanical coupling capabilities (*k*_p_∼40%), making it a highly promising alternative to toxic lead-based ceramics in orthopedic applications. The intrinsic piezoelectricity of KNN arises from the transition between its rhombohedral, tetragonal, and orthorhombic phases. Studies have demonstrated that electrically polarized KNN surfaces, regardless of whether they are positively or negatively charged, exhibit significantly enhanced protein adsorption, cell adhesion, and osteoblast proliferation compared to their unpolarized counterparts (Dubey et al., 2013; Chen et al., 2017; Yao et al., 2019). To precisely replicate the heterogeneous electrophysiological microenvironment of natural bone, which consists of interspersed piezoelectric collagenous and non-piezoelectric non-collagenous domains, researchers have engineered microscale piezoelectric zones (MPZs) on KNN ceramics using selective laser irradiation (Yu et al., 2017). This technique induces localized phase transitions, creating adjacent regions of high and low piezoelectricity. Both *in vitro* and *in vivo* evaluations using rabbit femoral condyle defect models revealed that these biomimetic MPZ-patterned KNN ceramics dramatically upregulated osteogenic markers, such as RUNX2 and alkaline phosphatase (ALP), and facilitated superior bone regeneration compared to pristine KNN and conventional hydroxyapatite.

### 3.2 Organic piezoelectric polymers

Organic piezoelectric polymers (Figure 2D) are characterized by their remarkable flexibility, excellent processability, and low elastic modulus, which closely matches that of human soft tissues and unmineralized osteoid.

Natural and biomolecular piezoelectric materials possess irreplaceable advantages in bone tissue engineering due to their exceptional biocompatibility, inherent cell-recognition sites, and ideal *in vivo* biodegradability. Protein macromolecules, such as collagen and silk fibroin, represent typical examples of this category. Collagen serves as the primary source of piezoelectricity in natural bone [*d*_14_ ≈ 1–2 pC/N)], which originates from its non-centrosymmetric triple-helix polypeptide structure composed of amino acids like glycine and proline (Zhou et al., 2016; Ferreira et al., 2012). Upon mechanical stress, the distortion of the hydrogen bond network leads to the displacement of internal charge centers, generating a macroscopic shear piezoelectric potential. Similarly, silk fibroin provides a piezoelectric response dependent on its highly ordered anti-parallel-sheet crystalline regions, while also exhibiting remarkable toughness and processability (Yucel et al., 2011). Polysaccharide materials, including cellulose and chitosan, also exhibit piezoelectric characteristics derived from the physical displacement of intramolecular and intermolecular hydrogen bond dipoles under mechanical loading. Furthermore, the piezoelectricity of fundamental biological molecules, such as monomeric amino acids and peptides, has recently garnered significant attention. For instance, non-centrosymmetric *β*-phase glycine crystals exhibit an extraordinarily high piezoelectric strain constant (up to 178 pC/N) (Guerin et al., 2018), and diphenylalanine peptides can self-assemble into highly ordered nanotube structures to generate a robust shear piezoelectric response (Basavalingappa et al., 2020). These natural biomolecules completely circumvent issues of immune rejection and long-term toxicity, serving as an ideal foundation for constructing next-generation bioresorbable orthopedic implants.

Synthetic piezoelectric polymers are distinguished by their excellent processability, high flexibility, and low elastic modulus that closely matches human soft tissues and unmineralized osteoid. PVDF (*d*_33_ between −20 and -30 pC/N) (Martins et al., 2014) and its copolymer, polyvinylidene fluoride-trifluoroethylene (PVDF-TrFE, *d*_33_ between −30 and -40 pC/N) (Kim et al., 2017), are currently the most extensively investigated synthetic piezoelectric polymers. Their piezoelectric performance is primarily attributed to the polar-crystalline phase featuring an all-trans conformation. Manufacturing techniques such as electrospinning, mechanical stretching, or high-voltage poling can further increase the -phase content and optimize dipole orientation, thereby achieving high piezoelectric outputs. Although PVDF demonstrates superior electrical performance and chemical stability, its inherent high hydrophobicity and non-biodegradability typically necessitate surface hydrophilic modification or compounding with biodegradable materials to meet the long-term clinical requirements of bone repair. In contrast, PLLA has emerged as a highly attractive alternative due to its complete biological degradability. The piezoelectricity of PLLA originates from the reorientation of C=O dipoles along its chiral helical main chain when subjected to shear forces (Sultana et al., 2017). Following the promotion of bone healing, PLLA can be gradually metabolized into water and carbon dioxide *in vivo*, entirely eliminating the risk associated with secondary removal surgeries. Additionally, polyhydroxyalkanoates, such as polyhydroxybutyrate (PHB) and poly(3-hydroxybutyrate-co-3-hydroxyvalerate) (PHBV), constitute a class of naturally degradable polyesters synthesized by microorganisms. Their shear piezoelectric constants are remarkably close to those of natural human bone, demonstrating immense potential for the fabrication of temporary bone tissue engineering scaffolds.

### 3.3 Piezoelectric composites

Piezoelectric composites have emerged as a highly promising strategy for bone tissue engineering by addressing the inherent limitations of individual materials, specifically the mechanical brittleness and potential cytotoxicity of inorganic ceramics, as well as the relatively low electrical output of organic polymers. By integrating high-performance inorganic nanoparticles, such as BaTiO_3_ or ZnO into flexible polymeric matrices like PVDF-TrFE, PLLA, or PHB, these composites achieve a synergistic balance of robust electromechanical coupling, tunable biodegradability, and mechanical compliance that closely matches natural bone (Figure 2E). The incorporation of these inorganic fillers not only acts as a primary source of piezoelectricity but can also induce the crystallization of the electroactive *β*-phase within fluoropolymers, thereby exponentially enhancing the overall piezoelectric coefficient of the scaffold (Deng et al., 2019). However, the severe agglomeration of inorganic nanoparticles within organic matrices due to dielectric mismatch remains a significant challenge. To address this, researchers frequently employ surface functionalization techniques, such as applying a polydopamine (PDA) coating onto BaTiO_3_ nanoparticles, which not only ensures homogeneous dispersion and strong interfacial adhesion but also scavenges excessive ROS to optimize the local osteoimmune microenvironment (Huang et al., 2024). Furthermore, the integration of conductive nanomaterials, including graphene oxide (GO), carbon nanotubes (CNTs), or silver (Ag) nanoparticles, into these binary systems constructs advanced ternary composites. These conductive dopants facilitate the formation of efficient internal charge transfer networks, significantly amplifying the piezoelectric output and imparting additional functionalities such as robust antibacterial activity. Ultimately, whether fabricated *via* electrospinning into nanofibrous membranes or additively manufactured into 3D porous scaffolds, these advanced piezoelectric composites successfully restore the endogenous electrophysiological microenvironment, thereby accelerating osteogenic differentiation, angiogenesis, and structural integration in critical-sized bone defects.

## 4 Emerging and frontier piezoelectric systems

While conventional bulk piezoelectric biomaterials possess robust inherent electromechanical properties, their translational utility in complex orthopedic settings is frequently bottlenecked by rigid structural limitations. For instance, pristine piezoelectric ceramics are plagued by severe brittleness, structural mismatch with cancellous bone, and non-degradability, whereas traditional synthetic films lack the essential three-dimensional porous architecture required to sustain space-making niches for osteoblast infiltration and vascular ingrowth. To bridge these gaps, contemporary orthopedic research has transcended passive material selection, pivoting toward advanced multi-dimensional structural formulations and smart responsive systems. These emerging paradigms encompass soft hydrogel networks, low-dimensional nanomaterials, and intelligent 3D or 4D-printed scaffolds (Figure 2F). They leverage cutting-edge soft matter engineering, nanotechnology, and advanced manufacturing to configure baseline piezoelectric components into highly biomimetic, geometry-adaptive, and programmable architectures capable of providing customized electromechanical cues synchronized with the dynamic stages of skeletal regeneration.

Among the frontier formulations engineered for minimally invasive orthopedics, piezoelectric hydrogels represent a highly attractive class of soft bio-energetic systems (Vinikoor et al., 2023; Choudhury et al., 2025). These hydrogels elegantly synergize the classic physiological advantages of hydrogel matrices, characterized by high water retention, open porous network structures, high nutrient diffusivity, and an elastic modulus mimicking unmineralized osteoid, with the robust biophysical stimulation offered by active electromechanical transducers. The general fabrication methodology involves the homogeneous encapsulation of electroactive nanopowders or self-assembled organic crystalline blocks into a hydrophilic covalently or physically crosslinked polymeric network (Choudhury et al., 2025). The structural polymer matrix typically comprises highly biocompatible natural polymers such as gelatin methacryloyl (GelMA), silk fibroin, hyaluronic acid, and sodium alginate, or cytocompatible synthetic blocks such as polyethylene glycol (PEG) and polyvinyl alcohol (PVA). Dispersed within these highly hydrated spaces, low-dose inorganic fillers (e.g., tetragonal BTO nanoparticles or ZnO whiskers) or biomolecular assemblies (e.g., diphenylalanine nanotubes) serve as the primary source of localized electrical dipole generation. Under external remote stimulation fields, most notably LIPUS, the acoustic pressure waves induce subtle elastic deformations within the surrounding soft hydrogel network. This mechanical strain propagates across the material-filler interface, displacing the internal charge centers of the embedded non-centrosymmetric nanoparticles to yield transient, localized surface potentials. From a clinical standpoint, these injectable formulations possess immense superiority when dealing with geometrically intricate, irregular bone defects caused by compound trauma or high-degree tumor resections. Rather than requiring extensive surgical exposures to fit pre-carved rigid blocks, these fluid precursors can be delivered directly into the target cavity *via* standard syringe injection, where they crosslink *in situ* to establish a conformable, seamless interface with the surrounding host bone.

Concurrently, the rapid evolution of nanotechnology has catalyzed the exploration of low-dimensional piezoelectric nanomaterials, encompassing zero-dimensional nanoparticles, one-dimensional nanowires, and two-dimensional nanosheets. Due to quantum confinement and nanoscale size effects, these low-dimensional structures exhibit significantly enhanced electromechanical coupling capabilities, ultra-high specific surface areas, and unique piezotronic or piezo-phototronic properties (Zhao et al., 2023). Two-dimensional materials, such as transition metal dichalcogenides and surface-modified graphene oxide, offer extreme flexibility and sensitivity, enabling precise intracellular interactions and the construction of self-powered, targeted nano-therapeutics. One-dimensional structures, including ZnO nanorods and BTO or KNN nanowires, possess extraordinarily high aspect ratios and mechanical sensitivity. At the cell-material interface, these nanoscale protrusions function as biomimetic structural mimics of native extracellular matrix fibrils. When adhering cells exert endogenous mechanical forces, including cell traction, focal adhesion spreading, or active cytoskeletal contraction, the resulting localized bending of these nanofibrils generates an instantaneous on-demand surface potential (Liu et al., 2021b; Xue et al., 2021). This mechanical interaction is capable of triggering localized depolarization of the cell membrane, selectively opening stretch-activated and VGCCs without requiring macroscopic skeleton deformation or external energy inputs (Zhang et al., 2024). Simultaneously, two-dimensional piezoelectric nanosheets, such as transition metal dichalcogenides (e.g., monolayer molybdenum disulfide, MoS_2_), surface-modified graphene oxide (GO), and electroactive transition metal carbides/nitrides (MXenes), have garnered substantial attention due to their supreme mechanical flexibility, atomic thickness, and high surface area (Liu et al., 2025). In these ultra-thin matrices, the broken inversion symmetry in the planar crystal lattice gives rise to strong, highly responsive piezoelectric polarization under minuscule in-plane shear strains. Furthermore, the immense surface area of 2D nanosheets provides an abundant landscape for chemical functionalization and coordinated elemental doping. This structural feature allows for the simultaneous delivery of trace osteogenic ions (e.g., Zn^2+^ and Mg^2+^) or the implementation of targeted piezocatalytic ROS generation cascades, rendering low-dimensional systems highly effective components for the design of smart, multi-functional bone tissue engineering scaffolds.

The evolution of additive manufacturing has transformed the fabrication of bone tissue engineering scaffolds from static morphological templates into dynamic, time-dependent interactive systems (Chopra et al., 2026). While traditional 3D printing enables the precise, layer-by-layer spatial arrangement of piezoelectric polymers and ceramic composites to replicate the custom anatomical geometry and macro-porosity of patient-specific defects, 4D bioprinting introduces a crucial temporal axis into the regenerative landscape. The core design principle of crelies on combining shape-memory polymers (SMPs, such as shape-memory polyurethanes, biodegradable polylactic-co-glycolic acid (PLGA) networks, or crosslinked polycaprolactone) with highly responsive piezoelectric nanoparticles (Chen A. et al., 2024). Upon printing into complex, porous structural configurations, these smart scaffolds can be temporarily compressed or deformed into a compact shape to facilitate minimally invasive surgical delivery. Once deployed at the target defect site, the exposure to specific physiological triggers, such as body temperature, or external remote energy fields like localized alternating magnetic fields or focused ultrasound, actuates the shape-memory network, driving a programmable macroscopic transformation back to its original pre-designed target geometry. Crucially, this stimulus-responsive shape recovery process is not merely a mechanical adjustment to conform to the evolving margins of the bone defect. As the polymer chains undergo rapid macrostructural reorientation and phase transition, the resulting physical displacement generates strong internal shear forces that act directly upon the embedded piezoelectric nanoparticles. This transient mechanical actuation triggers a strong, sustained burst of localized surface charges, effectively functioning as an electromechanical switch that modifies the biophysical microenvironment at the exact moment of implantation (Chen et al., 2023). This temporal coordination of morphological expansion and active electrical discharge provides an optimal biological cue that recruits host mesenchymal stem cells, stimulates early focal adhesion maturation, and regulates localized macrophage polarization, representing a paradigm shift toward intelligent, patient-customized orthopedic therapies.

## 5 Advanced actuation strategies for piezoelectric biomaterial systems

The therapeutic efficacy of piezoelectric biomaterials is fundamentally predicated on their role as energy transducers, converting mechanical energy into localized electrical cues to steer cellular behavior. Unlike active electronic implants that require internal power sources, these scaffolds are inherently passive, necessitating consistent mechanical deformation to recapitulate the endogenous electrophysiological microenvironment of natural bone. In accordance with Wolff’s Law, early research primarily focused on passive actuation derived from spontaneous physiological activities, such as locomotion, weight-bearing, or muscle contractions. However, these endogenous stimuli are often insufficient or inconsistent, particularly in clinical scenarios involving severe immobility, large-segment defects, or deep-seated fractures where natural mechanical loading is limited. By leveraging external energy fields, including ultrasound-mediated vibrations, remote magneto-electric coupling, and wearable mechanical stimulators, researchers can now achieve precise, on-demand, and wireless control over the electrical output of implanted scaffolds. This section provides a comprehensive overview of these evolving actuation paradigms, evaluating their underlying biophysical mechanisms and their transformative potential in orchestrating precision bone regeneration.

### 5.1 Endogenous actuation: harnessing physiological movements

Endogenous actuation represents the most intrinsically biomimetic strategy, directly harvesting mechanical energy from the host’s spontaneous physiological activities to power piezoelectric scaffolds. This self-powered approach is informed by decades of *in vivo* bone strain research, which has provided a detailed understanding of how bone adapts to mechanical loads. Although direct measurements in humans are often restricted by the inherent invasiveness and complexity of the procedures, which typically focus on accessible sites such as the anteromedial aspect of the tibia or the dorsal surface of the metatarsal, the resulting datasets provide a precise blueprint for the mechanical demands placed on piezoelectric implants. Based on these *in vivo* findings, the strain magnitude in the human tibia typically fluctuates within a range of 0–5,000 *με* during routine daily activities (Yang et al., 2011; Burr et al., 1996), while high-impact scenarios such as jumping or basketball rebounding can drive these values to approximately 9,000 *με* (Milgrom et al., 2000a). Beyond magnitude alone, the bone strain rate is a critical determinant of osteogenic response and the corresponding electromechanical output of piezoelectric matrices. Under standard exercise conditions, bone strain rates generally fall between 1,500 and 20,000 *με*/s (Milgrom et al., 2001a; Milgrom et al., 2003; Milgrom et al., 2001b), yet they can escalate to peak values as high as 58,000 *με*/s during vigorous activities (Milgrom et al., 2000b).

The translation of these macroscopic loads into internal tissue stress distributions has been extensively characterized through advanced computational modeling (Carter, 1987). Theoretical analyses indicate that the skeletal microenvironment is governed by a complex interplay of hydrostatic (dilatational) and octahedral shear (deviatoric) stresses. In mature joint environments, internal octahedral shear stresses typically range from 0.25 to 1.75 MPa, with cumulative shear peaks potentially exceeding 6.0 Mpa under high-load conditions. Simultaneously, subchondral bone regions are subjected to intermittent hydrostatic compression reaching magnitudes up to −5.06 MPa. In contrast, the stress environment within the early fracture callus is significantly more compliant, with values generally confined to 20–90 Kpa range. For the design of piezoelectric scaffolds, these localized stress histories provide the essential biophysical parameters required to optimize electrical output across varying anatomical sites and healing stages. For piezoelectric scaffolds, these high strain rates are particularly significant, as they induce more rapid polarization changes within the crystal lattice, thereby generating more potent and frequent electrical potentials to facilitate bone remodeling. By integrating piezoelectric materials with appropriate elastic moduli and high sensitivity, these macroscopic impact forces are efficiently converted into localized electrical signals that synchronize with the patient’s movement rhythm.

Beyond macroscopic movement, the piezoelectric response is further sustained by continuous muscle contractions and localized cellular traction forces. Even in a resting state, where external loading is absent, advanced biomimetic nanostructures such as piezoelectric PLLA nanofibers are capable of harvesting minute mechanical energy directly from cell-material interactions. Research has quantified that localized cellular traction forces, typically ranging from 1 to 15 nN, can generate a dynamic on-demand piezo-potential between 11 and 103 µV. This sensitive interaction ensures a baseline electrical output that maintains the electrophysiological activity of the bone niche by triggering intracellular calcium transients and upregulating key signaling proteins, such as calmodulin (Cam) and CaMKII. By promoting cell spreading and the maturation of focal adhesions, these adhesion-mediated signals facilitate osteogenic differentiation and accelerate tissue recovery even during periods of physical inactivity (Zhang et al., 2024).

Despite its autonomous nature and inherent biocompatibility, the clinical application of endogenous actuation is restricted by its uncontrollable stochasticity and variable intensity. The magnitude and frequency of the generated electrical signals are strictly coupled to the patient’s mobility and physical vigor, which presents a significant hurdle for those suffering from critical-sized bone defects or severe trauma. For bedridden individuals who lack sufficient endogenous mechanical dynamics, the internal environment remains electrically silent, often providing inadequate stimulation to drive accelerated bone regeneration. This inherent limitation necessitates the development of more controllable, exogenous actuation strategies that can provide robust and continuous electrical cues independent of the patient’s physical activity level.

### 5.2 Ultrasound-triggered actuation: from LIPUS to deep-tissue stimulation

LIPUS represents the most advanced remote actuation strategy for piezoelectric scaffolds, transforming standard mechanical therapy into a highly potent electromechanical intervention. LIPUS is a specific modality of mechanical energy delivered as periodic acoustic pressure waves at an intensity below 3 W/cm^2^ (Malizos et al., 2006; Zong et al., 2025). In standard clinical applications for bone healing, LIPUS typically operates at a medium frequency of 1.5 MHz (within the broader 0.7–3.0 MHz therapeutic range), 20% pulse duty cycle, 200 µs pulse width, 20 min of daily exposure, and a spatial-average temporal-average intensity of 30 mW/cm^2^ (Heckman et al., 1994; Yang et al., 2005; Schandelmaier et al., 2017; Jiang et al., 2018). Upon reaching the implanted piezoelectric scaffold, the periodic acoustic pressure induces micro-deformations within the piezoelectric crystalline lattice, generating a proportional electrical field. To maximize this *in situ* energy harvesting, scaffolds can be structurally optimized (*via* porosity and geometry) so that their inherent resonance frequency aligns with the applied ultrasound frequency (typically 1–3 MHz), achieving peak acousto-electric conversion efficiency.

**FIGURE 3:**
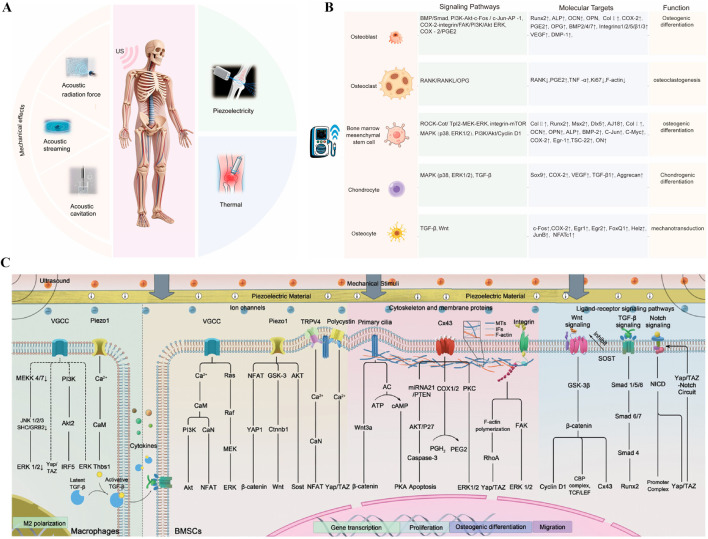
**(A)** Fundamental biophysical principles of LIPUS-mediated bone remodeling. The interaction between LIPUS and bone tissue results in three primary physical phenomena: mechanical actions (comprising acoustic radiation force, streaming, and cavitation), piezoelectric responses, and localized thermal change. Reproduced with permission (Zong et al., 2025). Copyright 2025, MDPI. **(B)** Intracellular signaling networks and molecular mediators through which LIPUS regulates the osteogenic, chondrogenic, and osteoclastic activities of bone-resident cells, alongside osteocyte mechanotransduction (Zong et al., 2025). Reproduced with permission (Zong et al., 2025). Copyright 2025, MDPI. **(C)** Schematic illustration of how piezoelectric stimuli accelerate bone formation, with dashed lines highlighting putative electrosensitive transducers within the cellular environment. Reproduced with permission (Zhang J. et al., 2025). Copyright 2025, Wiley-VCH.

LIPUS propagates as a mechanical wave, exerting inherent therapeutic effects on bone tissue primarily through mechanical effects [including acoustic radiation force (Gao et al., 2020; Jiang Z. et al., 2024; Menz et al., 2019), acoustic streaming (Wright et al., 2011; Xin et al., 2016; Uddin et al., 2011), and stable cavitation (Li et al., 2021; Xue et al., 2022; Shen et al., 2016)], piezoelectric effect, and thermal effects, as shown in Figure 3A. ARF generates micronewton-level periodic pressure that induces cellular displacement and vibrations, directly activating mechanosensitive receptors on the cell membrane. Acoustic streaming drives interstitial fluid flow, which not only accelerates nutrient delivery and waste removal but also exerts fluid shear stress to activate osteoblast mechanotransduction. Concurrently, stable cavitation of microbubbles produces localized microflows and shear forces without violent collapse. Together, these pure acoustic forces stimulate bone healing independently, providing a baseline mechanical stimulus before electromechanical conversion occurs.

Beyond these primary mechanical forces, LIPUS induces a distinct piezoelectric response as its acoustic pressure acts upon the non-centrosymmetric structures of collagen fibers and hydroxyapatite crystals (Fukada and Yasuda, 1957). This electromechanical coupling generates localized polarized charges that serve as an additional biophysical signal within the bone matrix (Suzuyama et al., 2023; Hosokawa, 2016a; Hosokawa, 2016b). Simultaneously, a subtle thermal effect arises from the dissipation of acoustic energy through viscous and relaxational losses in the surrounding tissue (Chen W. et al., 2024; Li et al., 2006). This process converts acoustic energy into heat *via* molecular friction, resulting in a marginal elevation of local temperature that can modulate cellular excitability (Song et al., 2023). Consequently, the comprehensive regulation of bone remodeling by LIPUS is achieved through the synergistic integration of mechanical, piezoelectric, and thermal stimuli. While mechanical effects remain the dominant factor at typical clinical intensities, the concurrent piezoelectric signaling and minor thermal cues provide sophisticated regulatory layers that optimize the overall cellular response.

Therefore, ultrasound acts on bone cells, such as osteoblast (Yang et al., 2005; Li et al., 2006; Miyasaka et al., 2015; Gleizal et al., 2006), osteoclast (Sun et al., 2001; Monici et al., 2007; Sun et al., 2021), bone marrow mesenchymal stem cell (Yang et al., 2019; Xie et al., 2019; Aliabouzar et al., 2018; Xia et al., 2017; Wang et al., 2019), chondrocyte (Wiltink et al., 1995; Ikeda et al., 2006; Mukai et al., 2005), and osteocyte (Fung et al., 2014; Shimizu et al., 2021), through various molecular and signaling pathway routes, as shown in Figure 3B. A core advantage of ultrasound is its exceptional penetration depth. Ultrasound waves can effectively travel through several centimeters of skin, fat, and muscle tissue with minimal attenuation and negligible thermal damage, precisely reaching deep-seated bone defects. This enables true non-invasive, *in situ* energy harvesting, completely eliminating the infection risks associated with transdermal wires or the need for bulky implanted batteries.

While LIPUS is already a clinical standard for fracture healing by providing mechanical vibration, its combination with piezoelectric scaffolds achieves a synergistic 1 + 1>2 effect through dual mechanical and electrical stimulation. Piezoelectric stimulation-induced osteoconductivity in stem cells primarily depends on voltage- and mechanosensitive ion channels, cytoskeletal proteins, membrane proteins, and ligand–receptor signaling pathway, as shown in Figure 3C (Zhang J. et al., 2025). For example, the mechanical strain directly activates mechanosensitive PIEZO1 channels, while the generated piezoelectric signals simultaneously open voltage-gated calcium channels, driving a massive intracellular influx. This synergistic calcium signaling robustly activates the CaM/CaMKII cascade, subsequently upregulating the transcription of early (COL1a, BMP-2) and late (RUNX2, OCN) osteogenic markers.

To systematically illustrate the paradigm shift from relying on endogenous mechanical forces to utilizing external ultrasound actuation, a comprehensive comparison of representative studies is presented in Table 1. This table clearly delineates the distinct material types, applied parameters, localized electrical outputs, and the profound biophysical mechanisms underlying bone regeneration under different actuation modes. By contrasting these two strategies, readers can better distinguish the specific biological effects and clinical potential of ultrasound driven piezoelectric signaling.

**TABLE 1:** Summary of representative piezoelectric studies actuated by endogenous mechanical forces *versus* external ultrasound stimulation.

Material type	Actuation mode and parameters	Piezoelectric output	Proposed mechanism	Biological outcome	References
Without ultrasound (endogenous mechanical)					
PMMA and 15 wt% BaTiO3 bone cement	Biomechanical stress (152 kPa, 0.65 Hz, 20 min)	Open-circuit voltage of 37.109 V	Activated calcium-sensitive receptors and increased intracellular calcium ion inflow	Significantly promoted osteogenic differentiation (upregulated ALP and RUNX2) and interfacial bone integration	Wang et al. (2024)
PLLA piezoelectric film	Exercise-induced joint movement (0.08 MPa pressure/33 N impact)	Output voltage of 3.6 V	Generated stable microcurrents to modulate cellular behavior	Enhanced cartilage gene expression and regeneration in rabbit knee joints	Liu et al. (2022c)
P(VDF-TrFE) nanoscaffold	Leg pulling/Muscle movement	6 mV, 6 nA	Surface charge generation	Stimulated osteoblastic activity *in vivo*	Wang et al. (2018)
BiFeO3-SrTiO3 implant	Physiological loads	+75 mV	Built in electric field	Enhanced osseointegration in bone defect walls	Liu et al. (2017)
Shape-memory-driven arch-shaped piezoelectric nanogenerator (sm-PENG)	Cyclic deformation	Peak current 20 µA	Promoted MC3T3-E1 preosteoblast cell proliferation, orientation and increase intracellular calcium ion	Increased calcium deposition, extracellular matrix mineralization and osteogenesis	Zhang et al. (2021)
PCL/liquid paraffin (HCBG)	Host muscle movement	40 V, 0.98 μA/cm^2^	Activated CaM/CaN/NFAT pathway	Promoted bone marrow stem cell ALP activity and regeneration	Yu et al. (2021)
Shape memory polyurethane elastomers/PVDF nanofibers	Shape recovery compressive force	Not specified	Initiated *in situ* voltage cues	Induced M2 macrophage polarization and increased bone volume	Li et al. (2025)
ZnO/Silk Fibroin hydrogel	Mechanical stress (0.1 MPa)	80 mV, 32 nA	Synergistic piezoelectric interactions	Enhanced osteogenesis and vascularization	Zhang et al. (2025c)
With Ultrasound (LIPUS or US-Driven)					
PLLA/BG/PAMAM-NH2 with Polyrotaxane	US activation (2.0 W)	Open-circuit voltage: 0.858 V, Short-circuit current: 3.516 µA	Activated Piezo1 channel and calcium/Calm-PI3K/Akt signaling pathway	Promoted robust osteoblast activity, RUNX2 expression, and bone regeneration	Zheng et al. (2025b)
Injectable silk fibroin/PVDF hydrogel	LIPUS (1 MHz, 100 mW/cm^2^ (pulsed wave)	Peak voltage of ∼80 mV	Activated NRF2 pathway to attenuate ROS and promoted endothelial mechanotransduction	Enhanced angiogenesis (VEGF/CD31 upregulation) and synergistic bone defect repair	Mo et al. (2026)
Tetragonal BaTiO3 NPs	US [1 MHz, 0.6 W/cm^2^	Not specified	Boosted ATP generation through OXPHOS	Reversed mitochondrial dysfunction and promoted PDLSC osteogenesis	Liu et al. (2024)
BaTiO3/multiwalled-carbon nanotubes/collagen membranes	LIPUS [1.5 MHz, 45 mW/cm^2^	0.93 pC/N	Ca^2+^ influx through Piezo1 channels	Induced M2 polarization and accelerated cranial bone repair	Jiang et al. (2024a)
KNN/nHA/PLA composite	LIPUS (1.5 MHz, 30 mW/cm^2^)	±75–80 mV, ±18–20 nA	PI3K/Akt mechanotransduction pathway	Comprehensive activation of osteogenic pathways (ALP, RUNX2, COL1)	Wang et al. (2025b)
PCL/BTO nanofilms	LIPUS (0.8 W/cm^2^)	Not specified	Modulated immune responses	Switched macrophages to M2 phenotype for tendon regeneration	Dai et al. (2025)
B-TNs@CMS (BTO in silk)	LIPUS (0.2 W/cm^2^)	Not specified	Modulated ROS generation *via* targeted piezocatalysis	Enhanced BMSC proliferation in diabetic microenvironments	Dong et al. (2025)
piezoTi (BaTiO3 coating on Ti)	LIPUS (1 W/cm^2^)	Not specified	Induced transient M1 status	Facilitated antibacterial capability and subsequent macrophage regulation	Li et al. (2023)
PLLA/MXene piezoelectric hydrogel	LIPUS (0.5 W/cm^2^)	Not specified	Induced ubiquitin-dependent mitophagy (PINK1-Parkin pathway) to enhance oxidative phosphorylation	Promoted M2 macrophage polarization to facilitate tissue regeneration	Zhang et al. (2026)
KZP@PS/PSPM (PLLA bilayer)	LIPUS stimulation	Not specified	Electrochemical reduction and stress responsive KGN release	Enhanced chondrogenesis and tendon-to-bone healing	Liang et al. (2025)
PLGA/Zn-KNN composite scaffold	US excitation (1 MHz, 1 m pulse width)	Open-circuit voltage of 40 mV	Upregulated immune responses and ion transmembrane transport	Enhanced macrophage phagocytosis against MRSA and regulated inflammatory cytokines	Zheng et al. (2025c)

## 6 Mechanisms of piezoelectric materials promoting osteogenesis

The endogenous electrical microenvironment is a fundamental biophysical regulator of bone homeostasis and tissue regeneration. Consequently, exogenous electrical stimulation (ES) has long been utilized in clinical settings to accelerate fracture healing by promoting osteoblast proliferation, enhancing angiogenesis, and modulating localized immune responses (Farjaminejad and Dingle, 2025). However, conventional ES typically relies on bulky, wired, or invasive power sources, which severely restricts its broader clinical implementation. To overcome these inherent limitations, piezoelectric biomaterials have emerged as a revolutionary, self-powered therapeutic strategy. By directly converting physiological mechanical loads into localized surface electrical charges, these smart materials perfectly replicate the natural electromechanical coupling of native bone without requiring external batteries. The underlying mechanism by which these mechanically induced piezoelectric signals promote osteogenesis is highly orchestrated. It involves a sophisticated biophysical and biochemical cascade that seamlessly spans from initial interfacial electrophysiological transduction to the ultimate structural integration and matrix mineralization of the newly formed bone.

### 6.1 Alteration of cell membrane potential and ion channel activation

**FIGURE 4:**
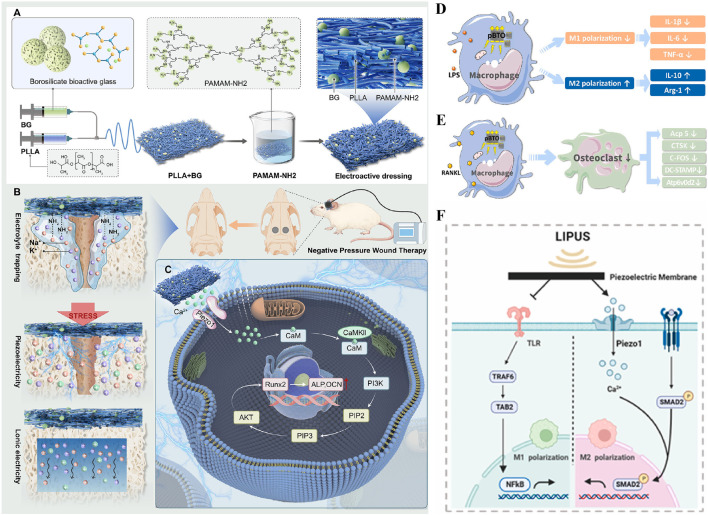
**(A)** Fabrication process of the PLLA/BG/PAMAM-NH_2_ electroactive dressing. Reproduced with permission (Zheng Z. et al., 2025). Copyright 2025, Wiley-VCH. **(B)** Mechanism of discharge and ion capture by the PLLA/BG/PAMAM-NH_2_ electroactive dressing. Reproduced with permission (Zheng Z. et al., 2025). Copyright 2025, Wiley-VCH. **(C)** Promotion of bone regeneration *via* coupling of internal and external electric fields, including the underlying molecular mechanisms. Reproduced with permission (Zheng Z. et al., 2025). Copyright 2025, Wiley-VCH. **(D)** Schematic diagram of intracellular wireless electric field induced by pBTO promote M2 polarization and inhibit M1 polarization. Reproduced with permission (Zuo et al., 2025). Copyright 2025, Elsevier. **(E)** Schematic diagram of pBTO inhibit osteoclastogenesis of macrophage. Reproduced with permission (Zuo et al., 2025). Copyright 2025, Elsevier. **(F)** Schematic summary of the macrophage polarization pathways induced by BMC + L. L, LIPUS stimulation. Reproduced with permission (Jiang T. et al., 2024). Copyright 2024, Elsevier.

Electrical stimulation serves as a potent biophysical cue that interacts directly with the cell membrane, altering the resting membrane potential to drive depolarization or hyperpolarization across various cell types involved in bone regeneration. Piezoelectric biomaterials provide localized, self-powered electrical signals that perturb the electrostatic field at the cell-material interface, serving as the primary biophysical contact point to activate specific transmembrane ion channels. For bone marrow mesenchymal stem cells (BMSCs), Zheng *et al.* developed an electroactive dressing based on piezoelectric poly(L-lactic acid) doped with bioactive glass and amino-terminated dendritic macromolecules (PLLA/BG/PAMAM-NH_2_), as shown in Figure 4A. Under negative pressure, it promoted massive Ca^2+^ influx, activating the PI3K/Akt signaling pathway to robustly enhance osteogenic differentiation (Figures 4B,C) (Zheng Z. et al., 2025). Similarly, in pre-osteoblasts, Xia *et al.* established a direct-current triboelectric nanogenerator that specifically activated the mechanosensitive transient receptor potential vanilloid 4 (TRPV4) ion channel, facilitating a targeted Ca^2+^ surge that upregulated osteogenic gene expression and matrix mineralization (Xia et al., 2025). Furthermore, these electrical fields exert a distinct regulatory effect on bone-resorbing cells. Zuo *et al.* demonstrated that endocytosed polarized BTO (pBT) nanoparticles generated an intracellular wireless nanoelectric field in osteoclast precursors that specifically upregulated the mitochondrial magnesium transporter MRS2; this drove mitochondrial Mg^2+^ influx and metabolic reprogramming, effectively inhibiting excessive osteoclastogenesis, as shown in Figures 4D,E (Zuo et al., 2025). The fluctuation of membrane potential and ion channel activation crucially extends to local immune cells as well. Jiang *et al.* engineered a biomimetic piezoelectric BTO/carbon nanotube/collagen (BMC) periosteum that, under ultrasound stimulation, specifically opened mechanosensitive PIEZO1 channels in macrophages; the resulting influx suppressed Toll-like receptor signaling to drive robust M2 polarization, successfully accelerating bone regeneration in cranial defects (Figure 4F) (Jiang T. et al., 2024). Finally, regarding vascularization, Mo *et al.* engineered an ultrasound-responsive piezoelectric silk fibroin/PVDF hydrogel that provides dynamic electromechanical cues to endothelial cells; these signals directly activate endothelial mechanotransduction pathways and promote cytoskeletal reorganization, thereby accelerating focal adhesion dynamics to facilitate rapid vascular network formation (Mo et al., 2026).

### 6.2 Reprogramming of cellular energy metabolism

The osteogenic differentiation of mesenchymal stem cells is a highly energy-demanding biological process that requires substantial metabolic support for intensive extracellular matrix synthesis and mineralization. Piezoelectric stimulation has emerged as a potent regulator of cellular bioenergetics by directly rejuvenating mitochondrial function. For instance, Liu *et al.* developed a piezoelectric hydrogel incorporating tetragonal barium titanate nanoparticles; under local mechanical stress, the generated piezopotential effectively restored the mitochondrial membrane potential and significantly increased intracellular adenosine triphosphate (ATP) synthesis in inflammatory periodontal ligament stem cells, successfully rescuing their impaired osteogenic differentiation capacity (Liu et al., 2024). Similarly, Wu *et al.* demonstrated that piezoelectric BaTiO_3_-coated Ti_6_Al_4_V scaffolds under dynamic loading significantly upregulated oxidative phosphorylation (OXPHOS) and ATP production in macrophages, which provided the essential metabolic basis for their anti-inflammatory polarization (Wu et al., 2023).

**FIGURE 5:**
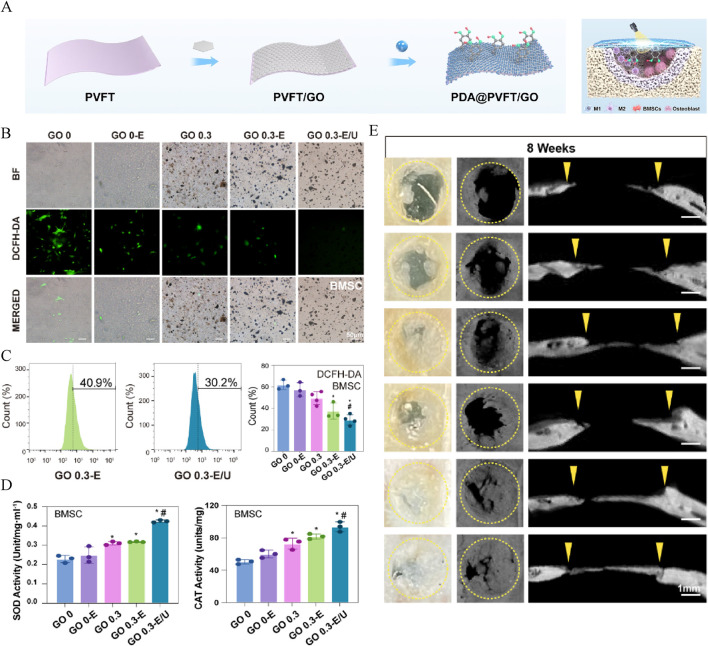
**(A)** Illustration of the fabrication workflow for the PDA@PVFT/GO piezocatalytic nanocomposite membrane. **(B)** Characteristic immunofluorescence images displaying ROS production (visualized in green) in BMSCs subjected to 48 h of hyperglycemic conditions, specifically 25 mM d-glucose. **(C)** Flow cytometric data and associated quantification of DCFH-DA signals in BMSCs following a 48-h incubation in high-glucose media. **(D)** Enzymatic activity levels of superoxide dismutase (SOD) and catalase (CAT) in BMSCs evaluated after a 48-h hyperglycemic challenge. **(E)** Clinical photographs and corresponding micro-CT reconstructions of critical-sized rat calvarial defects at 8 weeks post-implantation. Yellow arrows identify the transition zone between newly formed bone and the host tissue. Reproduced with permission (Sun et al., 2024). Copyright 2024, Elsevier.

Concurrently, piezoelectric stimulation plays a crucial role in maintaining cellular redox homeostasis. In trauma-induced or inflammatory bone defect microenvironments, excessive ROS often accumulate, inducing severe oxidative stress that impairs cell viability. To address oxidative challenges, Sun and colleagues engineered a polarized ferroelectric nanomembrane by functionalizing a graphene oxide/P(VDF-TrFE) matrix with a polydopamine (PDA) surface coating (PDA@PVFT/GO) (Figure 5A). The bioactive polyphenol moieties within the PDA layer serve as efficient scavengers for ambient ROS through redox pathways. Experimental evidence from DCFH-DA assays and flow cytometry confirmed that the synergy between the membrane and ultrasound stimulation significantly attenuated oxidative stress in both macrophages and BMSCs (Figures 5B,C). This protective effect was further substantiated by the upregulated expression of antioxidant enzymes, including SOD and CAT (Figure 5D). *In vivo*, micro-CT analysis at week 8 demonstrated that bone defects treated with these polarized composite membranes under daily US exposure were successfully replenished with mature bone (Figure 5E), exhibiting markedly superior bone volume fraction (BV/TV) and trabecular density compared to control cohorts (Sun et al., 2024). Developing antioxidant biomaterials to scavenge excessive ROS is a pivotal strategy for bone repair, as macrophage-induced oxidative stress significantly impairs osteogenesis. PDA coatings have emerged as effective agents to mitigate local ROS levels and facilitate M1-to-M2 macrophage polarization, thereby establishing a pro-healing microenvironment. For instance, Liu *et al.* engineered a PDA-functionalized biomimetic periosteum that demonstrated robust ROS-scavenging capacity in DPPH and flow cytometry assays. Quantitative 3D micro-CT imaging and BV/TV analysis further revealed that the PHA/5%PBT group achieved superior bone volume under piezoelectric stimulation. This enhanced regenerative performance is attributed to the synergistic effects of PDA-mediated cell adhesion and ROS scavenging, which suppress inflammation and promote anti-inflammatory M2 phenotypes to accelerate bone formation (Liu et al., 2023).

### 6.3 Remodeling of the osteoimmune microenvironment

**FIGURE 6:**
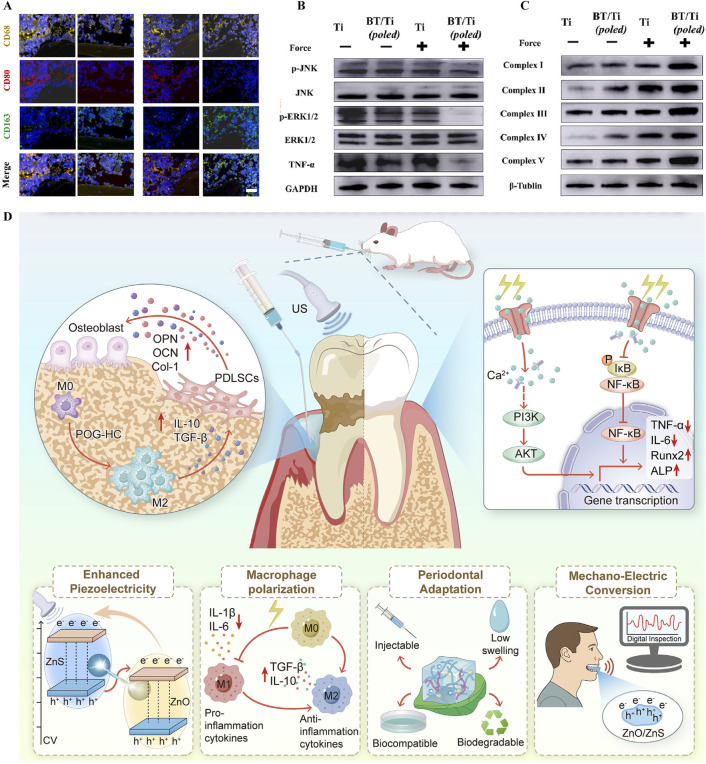
**(A)** Typical immunofluorescent micrographs identifying general (CD68^+^, yellow), pro-inflammatory M1 (CD80^+^, red), and pro-regenerative M2 (CD163^+^, green) macrophage populations within the peri-implant bone of sheep vertebral models. **(B)** Immunoblotting assessment of signaling cascades associated with inflammatory responses in RAW264.7 cells after 24 h of scaffold incubation. **(C)** Western blot evaluation of mitochondrial electron transport chain complex expression related to oxidative phosphorylation and bioenergetic output in RAW264.7 macrophages. Reproduced with permission (Wu et al., 2023). Copyright 2023, Elsevier. **(D)** Schematic representation of how the functionalized hydrogel promotes osteoimmune regeneration through enhanced piezoelectric performance and immune modulation. Reproduced with permission (Zhou et al., 2026). Copyright 2026, Wiley-VCH.

The successful integration and regeneration of bone implants heavily depend on the delicate orchestration of the osteoimmune microenvironment, which involves a dynamic crosstalk among various innate and adaptive immune cells. While persistent inflammation hinders bone healing, the electrical cues provided by piezoelectric biomaterials actively remodel this niche into a pro-regenerative state. Macrophages, as the primary effectors, exhibit remarkable phenotypic plasticity in response to these electromechanical signals. For instance, Wu *et al.* developed a piezoelectric BaTiO_3_-coated Ti_6_Al_4_V scaffold that profoundly reprogrammed macrophage behavior by modulating both inflammatory signaling and metabolic pathways. To evaluate the local immune response around the sheep artificial vertebral bodies, immunofluorescent staining was utilized to identify general (CD68), M1 (CD80), and M2 (CD163) macrophage subtypes (Figure 6A). The results demonstrated that poled BT/Ti implants fostered a more favorable regenerative environment, characterized by a significant reduction in the proportion of pro-inflammatory CD80^+^ M1 cells and a marked elevation in pro-healing CD163^+^ M2 macrophages compared to the pure Ti group. Mechanistically, the dynamic piezoelectric signals effectively inhibited the primary protein kinases of the MAPK cascade, resulting in the marked suppression of phosphorylated JNK, ERK, and TNF-α (Figure 6B). Concurrently, the stimulation enhanced the expression of all main electron transport chain complexes (Complex I–V, Figure 6C), which significantly promoted basal respiration and ATP synthesis during oxidative phosphorylation (OXPHOS) while distinctly inhibiting glycolysis. This metabolic reprogramming robustly drove macrophage polarization toward the pro-healing M2 phenotype. *In vivo* immunofluorescence evaluations in a sheep cervical defect model visually confirmed this shift, demonstrating a significant accumulation of CD163^+^ M2 macrophages and a marked reduction of CD80^+^ M1 macrophages in the bone tissues surrounding the piezoelectric implants (Wu et al., 2023). Similarly, Zhou *et al.* engineered a multifunctional piezoelectric hydrogel incorporating ZnO/ZnS heterojunctions; upon ultrasound activation, the generated electrical signals significantly suppressed the secretion of pro-inflammatory cytokines (TNF-α and IL-6) while upregulating IL-10 and TGF-β, robustly driving M2 macrophage polarization to restore alveolar bone architecture in inflammatory periodontitis (Zhou et al., 2026). These M2 macrophages subsequently secrete abundant regenerative cytokines and growth factors, including BMP-2, TGF-α, and VEGF, which synergistically accelerate mesenchymal stem cell recruitment, osteoblast differentiation, and angiogenesis (Figure 6D) (Vats et al., 2006).

Beyond macrophages, piezoelectric signals critically regulate other key immune populations. During the early acute inflammatory phase, He *et al.* engineered a piezoelectric ceramic nanofiber aerogel incorporating (K,Na)NbO_3_ that, under ultrasound activation, successfully redirected neutrophils from highly inflammatory pyroptosis toward immunologically silent apoptosis; this process simultaneously enhanced macrophage efferocytosis to rapidly resolve chronic inflammation (He et al., 2026). Furthermore, these electromechanical cues extend their regulatory effects to the adaptive immune system. Dai *et al.* demonstrated that piezoelectric polycaprolactone/barium titanate (PCL/BTO) nanofilms significantly downregulated the Th17 cell differentiation pathway, reducing the local proportion of IL-17-producing CD3^+^ T cells and inhibiting the IL-17A/NF-B signaling axis to mitigate excessive immune-mediated tissue damage (Dai et al., 2025). Ultimately, this comprehensive immunomodulation establishes a highly favorable and balanced microenvironment for accelerated tissue regeneration.

### 6.4 Osteogenic gene transcription and matrix mineralization

**FIGURE 7:**
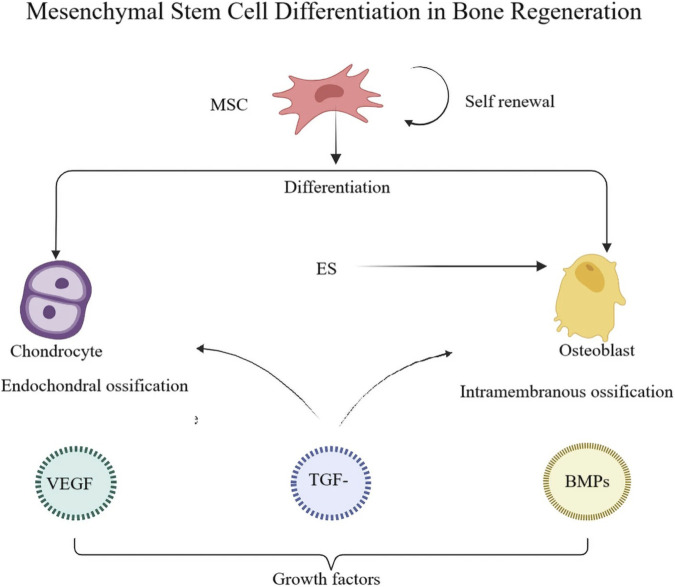
Conceptual diagram of mesenchymal stem cell fate determination during the bone healing process. These progenitor cells undergo either self-renewal or lineage-specific differentiation into chondrocytes and osteoblasts, thereby mediating endochondral and intramembranous ossification pathways, respectively. Key regulatory molecules, including VEGF, TGF-β, and BMPs, orchestrate these cellular transitions. Furthermore, electrical stimulation serves to augment osteogenic signaling cascades and facilitate the synthesis of the bone extracellular matrix. Reproduced with permission (Farjaminejad and Dingle, 2025). Copyright 2025, Springer Nature.

Bone regeneration is a complex, tightly regulated process that restores skeletal integrity through three overlapping phases: inflammation, repair, and remodeling. As illustrated in Figure 7, mesenchymal stem cells (MSCs) serve as fundamental precursors that respond to local growth factors (such as VEGF, BMPs, and TGF-β) to drive bone repair, osteogenesis, and angiogenesis (Ottewell, 2016). Throughout this process, bone formation and resorption are meticulously balanced through the RANKL/OPG pathway; osteoclasts resorb mineralized bone while osteoblasts drive new matrix deposition (Chen et al., 2018a; Boyce and Xing, 2008). The culmination of these biological events—along with upstream piezoelectric-induced ion channel activation, metabolic reprogramming, and immunomodulation—is the robust activation of osteogenic gene transcription. Intracellular signaling cascades ultimately converge on the nucleus to activate master transcription factors, notably RUNX2 and Osterix (Osx), which are further stimulated by TGF-β and BMPs (Wang et al., 2013). RUNX2 acts as the primary initiator, committing MSCs to the mature osteoblast lineage. Following transcriptional activation, osteoblasts secrete essential matrix proteins, including type I collagen (Col-1), ALP, osteopontin (OPN), and osteocalcin (OCN), to form the mineralization scaffold (Chen et al., 2018b). ALP plays a critical role in early mineralization by providing inorganic phosphate for hydroxyapatite crystal formation, while Col-1 provides the necessary structural framework for calcium deposition (Selvaraj et al., 2024).

Several recent studies have explicitly demonstrated this piezoelectric-driven transcriptional and mineralization cascade. For instance, Zheng *et al.* engineered an electroactive poly(L-lactic acid)/bioactive glass dressing that, under mechanical deformation, significantly upregulated the transcription of RUNX2 and Osx; this subsequently increased the expression of downstream ALP, OCN, and OPN, ultimately driving massive matrix mineralization to accelerate bone defect healing (Zheng Z. et al., 2025). Similarly, Wang *et al.* developed an ultrasound-responsive potassium sodium niobate/nano-hydroxyapatite/polylactic acid (KNN/nHA/PLA) composite scaffold; upon ultrasonic activation, the generated piezoelectric signals profoundly upregulated RUNX2 and COL1 protein expression, leading to extensive, mature calcium nodule deposition and advanced ECM mineralization (Wang Y. et al., 2025). Furthermore, Mo *et al.* utilized an injectable ultrasound-responsive silk fibroin/PVDF piezoelectric hydrogel to confirm this terminal regenerative phase; the dynamic electromechanical cues significantly enhanced the expression of RUNX2, Col-1, and OCN, synergistically amplifying extracellular matrix mineralization and successfully restoring biomechanical integrity in critical-sized femoral defects (Mo et al., 2026). Ultimately, this continuous cycle of targeted gene transcription and biomineralization ensures the successful functional integration of the implant and the complete restoration of native bone architecture.

### 6.5 Dual regulation and balancing of reactive oxygen species

Reactive oxygen species play a dual and highly context dependent role in bone tissue engineering. To address the complex physiological requirements of infected bone defects, it is crucial to clearly distinguish the two primary ROS regulation strategies employed by piezoelectric biomaterials, as shown in Table 2.

**TABLE 2:** Comparison of reactive oxygen species regulation strategies in piezoelectric biomaterials.

ROS strategy	Mechanism	Biological effects	Primary applications
ROS Generation	Ultrasound drives charge separation to generate abundant superoxide and hydroxyl radicals	Disrupts bacterial membranes induces lipid peroxidation and causes intracellular protein leakage	Antibacterial therapy and biofilm eradication
ROS Scavenging	Built in antioxidants or enzyme mimicking properties neutralize excess endogenous radicals	Alleviates oxidative stress protects stem cells and promotes M2 macrophage polarization	Anti inflammatory therapy and bone regeneration
ROS Balancing	Transient ultrasound generates ROS for sterilization followed by sustained antioxidant scavenging of residual ROS	Synchronizes rapid bacterial clearance with the restoration of a favorable osteoimmune microenvironment	Infected bone defect repair and complex tissue regeneration

On one hand, piezocatalytic ROS generation is primarily utilized for its potent antibacterial activity. Under mechanical stimulation such as ultrasound, piezoelectric materials undergo polarization to facilitate efficient electron and hole separation. These charge carriers react with surrounding oxygen and water to produce abundant oxidative radicals. This intense oxidative stress physically disrupts bacterial cell membranes, causes intracellular protein leakage, and effectively eradicates drug resistant biofilm infections. For instance, Guo and his team engineered magnetic and piezoelectric barium iron titanate nanoparticles. Under ultrasound stimulation, these nanoparticles effectively penetrate biofilms and generate ROS to physically disrupt bacterial membranes, successfully eliminating drug resistant bacteria (Wu et al., 2024). Additionally, Feng and colleagues utilized molybdenum disulfide piezoelectric nanomaterials. Driven by ultrasonic mechanical forces, these materials rapidly induce intense oxidative stress to eradicate deep tissue infections (Feng et al., 2022). Furthermore, Wang and coauthors designed defect rich heterojunction coatings to accelerate charge transfer under ultrasound. This design leads to a burst release of reactive oxygen species that achieves exceptional antibacterial efficiency against infectious pathogens (Wang C. et al., 2025).

On the other hand, ROS scavenging is essential for robust osteogenesis and immunomodulation (Soe et al., 2026). In the trauma microenvironment, excessive endogenous reactive oxygen species induce severe oxidative stress, which impairs stem cell viability and delays bone regeneration. To counteract this, piezoelectric biomaterials are often integrated with antioxidant components like polydopamine or designed to exhibit enzyme mimicking behaviors. This scavenging effect actively neutralizes excessive radicals, protects stem cells, and crucially drives macrophage polarization from a proinflammatory M1 phenotype toward a proregenerative M2 phenotype.

To bridge these two divergent needs, a new insight termed smart reactive oxygen species balancing or the reactive oxygen species lever approach has recently emerged. This advanced paradigm leverages transient ultrasound irradiation to generate reactive oxygen species for rapid bacterial clearance, followed by the continuous scavenging of residual reactive oxygen species *via* built in antioxidants once the ultrasound ceases. This dynamic synergy successfully eradicates infections while subsequently establishing a favorable osteoimmune niche for tissue repair. For example Geng and colleagues engineered an intelligent piezoelectric nanocatalytic heterojunction incorporating poly lactic co glycolic acid black phosphorus and vanadium carbide MXene. This advanced membrane exhibits properties similar to macrophages to combat antibiotic resistant infections. Crucially it successfully synchronized the production and elimination of reactive oxygen species highlighting the tremendous clinical potential of piezoelectric biomaterials (Geng et al., 2024). Similarly Wang and coworkers propelled this field forward by fabricating lithium doped zinc oxide and poly l lactic acid piezoelectric microfibers. These fibers were coated with the antioxidant 4 octyl itaconate for the targeted therapy of bacterially infected wounds demonstrating the significant positive impact of antioxidant coatings on reactive oxygen species balancing (Wang X. et al., 2025). Collectively these studies illustrate that piezoelectric materials equipped with appropriate antioxidant modifications can generate reactive oxygen species for precise sterilization while simultaneously exhibiting robust capabilities to scavenge excessive radicals under specific physiological conditions. This dual functionality completely transforms the traditional perception of piezoelectric materials in medicine and paves new avenues for their advanced application in biomedical therapies.

It's worth noting that the underlying mechanism by which mechanically induced piezoelectric signals promote osteogenesis is thought to involve a complex biophysical and biochemical cascade. While recent studies have reported significant upregulation of osteogenic markers following piezoelectric stimulation the precise causal relationships remain an active area of investigation. Rather than directly driving bone formation these smart biomaterials are proposed to modulate the local electrophysiological microenvironment. For instance the generation of localized surface electrical charges is associated with the activation of mechanosensitive ion channels and subsequent intracellular calcium transients. Furthermore instead of claiming that these materials robustly activate specific signaling cascades it is more accurate to state that piezoelectric stimulation may contribute to the regulation of key pathways based on current gene and protein expression profiling. Future studies employing specific pathway inhibitors and gene knockout models are required to fully validate these proposed mechanisms.

## 7 Conclusion and future perspectives

Piezoelectric biomaterials provide a revolutionary self-powered strategy for bone tissue engineering by replicating the native electromechanical microenvironment of natural bone. By converting endogenous physiological movements or exogenous stimuli like ultrasound into localized electrical signals, these smart materials effectively regulate crucial cellular behaviors. This biophysical stimulation alters cell membrane potentials, activates mechanosensitive and voltage gated ion channels, reprograms cellular energy metabolism, and modulates the osteoimmune microenvironment toward a pro healing state. Consequently, this orchestrated cascade significantly accelerates osteogenic gene transcription and extracellular matrix mineralization to facilitate robust bone regeneration. Despite these tremendous advancements, several critical limitations impede their immediate clinical translation.

### 7.1 Long term stability and degradation

The complex physiological environment poses a severe challenge to the long term stability of piezoelectric performance. The infiltration of water molecules and free ions in body fluids can disrupt hydrogen bonding networks and asymmetrical dipole alignment, severely suppressing the piezoelectric response. Furthermore, during long term dynamic loading, changes in the scaffold structure due to degradation may alter the spatial distribution and intensity of the electrical signals. Additionally, it remains unclear whether the deposition of calcium phosphate on the scaffold surface interferes with the transmission of mechanical stimuli to the piezoelectric material.

### 7.2 Standardization of stimulation parameters

Current research severely lacks standardized evaluation protocols and stimulation parameters. The extreme variability in applied intensity, frequency, duration, and device architecture complicates the accurate comparison of outcomes across different studies. Establishing a minimum reporting standard for electrical doses specifically at the tissue device interface is urgently required.

### 7.3 Distinguishing piezoelectric effects

It remains highly challenging to isolate genuine piezoelectric responses from confounding factors. Piezoelectric materials often introduce concurrent changes in surface topography, stiffness, and surface chemistry. Consequently, it is difficult to definitively distinguish pure piezoelectric effects from parallel mechanical or biochemical cues, especially when characterizing surface charges amidst electrostatic artifacts in complex liquid environments.

### 7.4 Barriers toward clinical translation

The transition from laboratory research to clinical application is hindered by significant translational barriers. These include stringent regulatory hurdles, the difficulty of achieving scalable and reproducible manufacturing for patient specific defects, and a heavy reliance on small rodent models. Future studies must prioritize long term evaluations in load bearing large animal models to accurately predict clinical efficacy and safety.

To bridge the gap between laboratory research and clinical application, future efforts should focus on several advanced directions. Regarding material innovation, research must prioritize the development of lead-free biodegradable hybrid composites that break down in stages, balancing electromechanical efficiency with safe degradation kinetics without releasing harmful byproducts like excessive reactive oxygen species or toxic nanoparticles. Researchers should stop viewing these materials as static frames and instead design dynamic environments, such as incorporating ultrasound sensitive particles inside soft hydrogels or utilizing self-assembling peptides that guide bone growth without interfering with cell movement.

The integration of artificial intelligence and molecular dynamics simulations represents a crucial future paradigm. Developing multidimensional molecular models that account for water infiltration and complex dipole interactions will shift piezoelectric biomaterial development from a trial and error approach toward precise, on demand molecular design. Machine learning models can be trained to predict functional piezoelectric coefficients based on structural and compositional data, significantly accelerating material discovery.

Clinically, the future lies in the creation of intelligent closed loop feedback systems. By incorporating flexible bioelectronics and biosensors, next-generation wearable or implantable devices could continuously monitor physiological indicators in real time. These smart systems would use artificial intelligence to automatically adjust the electrical stimulation parameters to match the specific developmental stages of bone healing.

Furthermore, to deepen our fundamental understanding, advanced methodologies like transcriptomics and proteomics must be utilized to comprehensively decode the precise molecular pathways bridging electrical stimulation and cellular signaling. Finally, establishing standardized testing guidelines is imperative. By combining piezoelectric materials with cutting edge tools like microfluidics, bioreactors, and organ on a chip technology, researchers can construct complex functional bone organoids equipped with vascular and neural networks, ultimately paving the way for personalized medicine and precise drug screening.
